# Low Vitamin D Concentration Is Associated with Increased Depression Risk in Adults 20–44 Years Old, an NHANES 2007–2018 Data Analysis with a Focus on Perinatal and Breastfeeding Status

**DOI:** 10.3390/nu16121876

**Published:** 2024-06-14

**Authors:** Victoria Rose Barri Benters Hollinshead, Julia L. Piaskowski, Yimin Chen

**Affiliations:** 1 MD/MPH Program, Southern Illinois University School of Medicine, Springfield, IL 62702, USA; vhollinshead31@siumed.edu; 2 College of Agricultural and Life Sciences, University of Idaho, Moscow, ID 83844, USA; jpiaskowski@uidaho.edu; 3 Margaret Ritchie School of Family and Consumer Sciences, University of Idaho, Moscow, ID 83844, USA

**Keywords:** 25OHD2, 25OHD3, pregnant, postpartum, women, diet patterns, mental health, PHQ-9, depressive symptoms, human milk

## Abstract

The objective was to investigate associations of serum vitamin D concentration with depressive symptoms and assess the impact that vitamin D concentration has on the occurrence of depressive symptoms in 20–44-year-old pregnant women, postpartum women, non-pp women (non-pregnant/postpartum women), and men, including a separate subgroup analysis of postpartum breastfeeding and non-breastfeeding women. The study populations were selected from the 2007–2018 NHANES public data. Subjective interview data and objective laboratory data including depressive symptoms, serum vitamin D concentration, nutrient intake, and demographic information were utilized. Two diet patterns were created using principal component analysis, and a Bayesian multinomial model was fit to predict the depression outcomes for each subpopulation. The estimates for the log vitamin D slope parameter were negative for all cohorts; as vitamin D increased, the probability of having no depression increased, while the probability of depression decreased. The pregnant cohort had the steepest vitamin D slope, followed by postpartum women, then non-pp women and men. Higher vitamin D concentration had more impact on decreasing depression risk in pregnant and postpartum women compared to non-pp women and men. Among postpartum women, higher vitamin D concentration had a greater influence on decreasing breastfeeding women’s depression risk than non-breastfeeding women.

## 1. Introduction

Women are at greater risk for major depression compared to men worldwide, and the causes of this discrepancy are still being investigated [1]. Within the U.S., data from the 2013–2016 National Health and Nutrition Examination Survey (NHANES) showed an almost 2:1 sex ratio difference in depression prevalence [2]. Biological factors, as well as environmental influences, may contribute to these differences [1]. In addition to being at higher risk based on gender, some women also experience perinatal depression-related illnesses which may impact the health of mothers and their infants [3,4,5,6,7]. Notably, antepartum depression has been associated with increased risk of preterm labor [3,5,7], preeclampsia [5], reduced fetal development [3,5], miscarriage [5], cesarean sections and instrumental vaginal deliveries [3], and admission to a neonatal care unit [3]. Infants whose mother experienced antepartum depression are also at higher risk of having low-birth weight and stunted growth across the first year of life [5]. Furthermore, the literature indicates a correlation between postpartum depression and child behavioral issues [4], impaired motor and cognitive development [6], infant sleep disturbances [6], and difficulties with mother–infant bonding [6,8]. 

Emerging studies are seeking to evaluate the relationship between vitamin D concentration and mental health [9,10,11,12]. Vitamin D has several roles in the body namely related to calcium homeostasis, bone health, immune function, gene expression, and neural function [9,13,14]. As such, vitamin D deficiency may contribute to a myriad of diseases, including depression [9,13]. One hypothesis examines how vitamin D decreases the heightened neuronal levels of calcium that propel depression [13]. Although the mechanism of how vitamin D contributes to depression remains unclear, correlations between low vitamin D concentration and depressive symptoms have been established in both men and women [9] as well as, specifically, pregnant [11,12,15] and postpartum women [10,15]. Notably, to the research team’s knowledge, no study has analyzed breastfeeding status in conjunction with these factors.

The human body acquires vitamin D through dietary intake and endogenous synthesis from adequate sun exposure. Poor dietary patterns and reduced sunlight exposure may consequently lead to vitamin D insufficiency/deficiency. In the United States, the National Center for Health Statistics showed that from 2001 to 2006, women had a greater risk of vitamin D deficiency compared to men [16], and another study reported a higher vitamin D concentration in men compared to women worldwide [17]. Particularly, women of childbearing age exhibit increased risk of vitamin D deficiency compared to men, but pregnant or lactating women have decreased vitamin D deficiency risk compared to other women [16]. Excluding the elderly population in countries with more supplement use, men have been shown to have increased vitamin D intake through nutrients and supplements compared to women [18]. To ensure the maintenance of adequate maternal and fetal/infant health, increased dietary and supplemental vitamin D intake is recommended in expecting mothers [19].

Many disparities exist in the U.S. maternal population with minority groups and lower socioeconomic status women experiencing higher rates of depression during pregnancy [5]. The social determinants of health contribute to the well-being of family units and involve factors such as education access, food security, work and home environment, healthcare access, financial stability, and quality of support systems. The social determinants of health are interconnected, impacting the physical and mental health of communities, and therefore they may influence the ability of mothers to exclusively breastfeed. Additionally, maternal mental health can not only impact maternal behaviors with the infant but also human milk composition. The literature shows that maternal nutrition [20] and stress levels [21,22] are associated with alterations in human milk composition. For example, Ziomkiewicz et al. found a positive correlation between stress reactivity and milk fat, long-chain unsaturated fatty acids, with a negative correlation between stress reactivity and milk lactose [22]. With this in mind, infants of different feeding practices and home-life environments could be receiving distinct nutrients from one another, contributing to their overall health. Due to potential changes in milk composition, the impact of maternal vitamin D concentration and postpartum depression may have health implications for infants who are breastfed compared to formula-fed. 

**Figure 1:**
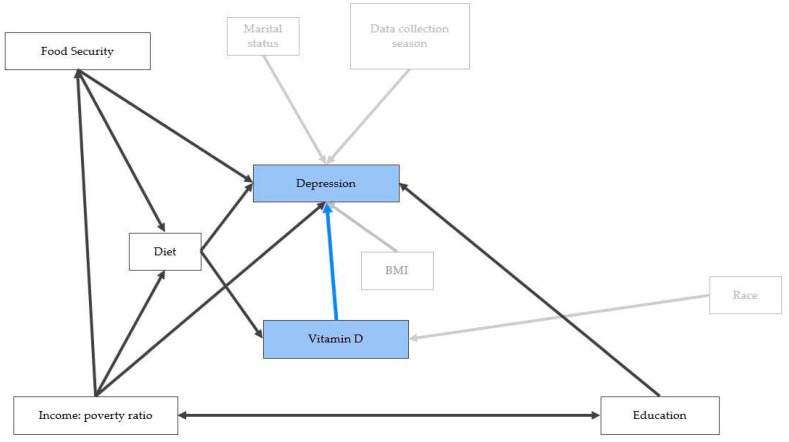
Directed acyclic graph displaying hypothesized relationships between key variables obtained from NHANES 2007–2018 data. Blue boxes: the study’s main variables of focus; white boxes: other key variables that have influences on the main variables; blue arrow: the study’s hypothesis of vitamin D concentration impacting depressive symptoms; bolded black arrows: variables that were shown in the literature review to influence more than one factor; faded gray arrows: variables that were shown in the literature review to influence one factor.

Depression is a complex medical condition caused or influenced by many factors. Figure 1 shows the directed acyclic graph created to visualize the relationships between vitamin D, depression, and other intervening factors that can impact both depression and vitamin D concentration and hence are possible confounding variables. Diet is a confounding factor, influencing both depression [23,24,25] and vitamin D concentration [14]. Additionally, diet is a mediating factor for the influence of other variables on depression and vitamin D concentration. For example, food security status has a negative correlation with depression status [26,27,28]. We did not find evidence in the literature that food security is directly influencing vitamin D concentration, but food security was shown to influence diet [29,30] and thus can indirectly impact vitamin D concentration. The income-to-poverty ratio may influence a person’s food security [30] and diet [31], providing secondary influences to depression and vitamin D concentration. Income-to-poverty ratio and education attainment can both influence one another; generational financial status has implications on educational achievements [32], while educational achievement further impacts income opportunity. A negative correlation between education level and depression was also identified in the literature [33,34]. Marital status, race, and data collection season were not found to be confounding factors. Instead, marital status [35,36] and season [37] were associated with depression, while race was correlated with vitamin D concentration [38,39,40]. Body mass index (BMI) has been positively correlated with depression [41], and despite negative correlations seen in the literature with BMI and vitamin D concentration [38,42,43,44], it was not included in the analyses as a confounding variable due to the complexity of influencing factors on BMI, including diet, physical activity, stress, genetics, environment, and more [45]. 

Women are at greater risk for depression than men, and some women experience maternal-related impacts on their mental health affecting both themselves and their infants. Vitamin D concentration and depression have been shown to have a negative correlation in several studies, and further investigations of this relationship are imperative to understanding the populations at greatest risk and the health interventions needed. The objective of this study was to investigate associations of serum vitamin D concentration with depressive symptoms and assess the impact that serum vitamin D concentration has on the occurrence of depressive symptoms in pregnant women, postpartum women, non-pp women (non-pregnant/postpartum women), and men with a subgroup analysis of postpartum breastfeeding and non-breastfeeding women. We hypothesize that serum vitamin D concentration will be negatively correlated with depression risk in all subpopulations, and higher vitamin D concentration will more strongly impact participants in the perinatal period. We also hypothesize that breastfeeding women will exhibit a lower amount of depression compared to other groups. Although previous studies have evaluated the relationship between vitamin D concentration and depressive symptoms, this study uses an extensive length of data (12 years; NHANES 2007–2018), comparing the four study groups of interest with each other, and incorporates a separate analysis of breastfeeding status in postpartum women, which together contribute unique information to better understand maternal and fetal health.

## 2. Materials and Methods

### 2.1. Study Populations

The National Health and Nutrition Examination Survey (NHANES) is a U.S. nationally representative series of cross-sectional, continuous surveys that combines interview questionnaires with physical exams and laboratory data collection. Open access data to the public are published every two years by the National Center for Health Statistics in conjunction with the Centers for Disease Control and Prevention. The NHANES participants are randomly selected for recruitment through analyses of U.S. Census information and provide informed consent prior to participation in the study. Approval of the survey cycles are permitted by the National Center for Health Statistics Research Ethics Review Board. The NHANES 2007–2018 survey cycles were chosen for this project because serum vitamin D concentrations were measured using the same method throughout the 12-year period. 

This study sought to evaluate how pregnant women, postpartum women, non-pp women (refers to women who are neither pregnant nor within 12 months postpartum), and men compare to each other with a separate, subset analysis of postpartum breastfeeding and non-breastfeeding women. Pregnancy status was determined through the NHANES demographic pregnancy status variable, which included urine pregnancy test results and/or self-reported status. Pregnancy status information is available for participants aged 20–44 years old; for equal comparative purposes, the study strictly analyzed participants within this age range for all study populations, and participants outside 20–44 years old were removed from this study. The research team defined postpartum women as having delivered 0–12 months before the time of the interview, which was presented by the ‘months since last delivery’ question in the reproductive health section. Men were separated from the other study populations through the gender/sex assessment (male or female) in the demographics section. The breastfeeding and non-breastfeeding women were evaluated separately as a subset of the postpartum group. The question assessing current breastfeeding activity was provided in the reproductive health section. 

### 2.2. Patient Health Questionnaire

Trained interviewers administered a nine-item depression screening instrument (Patient Health Questionnaire, PHQ-9) to assess self-reported depression symptoms over the previous two weeks, one symptom per question. Participants with missing PHQ-9 data were dropped from this study. The interviewers utilized the Computer-Assisted Personal Interview system throughout this portion of the Mobile Examination Center Interview. The questionnaire incorporates the fourth edition of the *Diagnostic and Statistical Manual of Mental Disorders* by scoring each of the symptom criteria [46]. Each question presented a symptom frequency scale ranging from 0 to 3 that corresponded with the responses 0 = “not at all”, 1 = “several days”, 2 = “more than half the days”, and 3 = “nearly every day”. These scores were summed across all 9 questions, resulting in a final PHQ-9 depression score that could range from 0 to 27. The PHQ-9 scores represent the level of depression severity: minimal, 0–4; mild, 5–9; moderate, 10–14; moderately severe, 15–19; and severe, 20–27 (46). The moderate (10–14), moderately severe (15–19), and severe (20–27) categories were combined into one category to balance the sample size across the depression groups, creating three final categories in the analysis: no depression (0–4), mild depression (5–9), and moderate to severe depression (10–27).

### 2.3. Serum Vitamin D Concentrations

Serum samples in NHANES 2007–2018 were evaluated by liquid chromatography–tandem mass spectrometry to quantify total serum vitamin D (25OHD2 + 25OHD3) with no changes in laboratory method, lab equipment, or lab site across each of the survey cycles. Participants with missing serum vitamin D concentration data were removed from this study.

### 2.4. Nutrient Intake within the Past 24 h

The What We Eat in America questionnaire was used in NHANES 2007–2018 for estimating dietary intake [47]. Total nutrient intake within the past 24 h of energy (kcal), protein (gm), carbohydrate (gm), total sugars (gm), total fat (gm), total saturated fatty acids (gm), and total monounsaturated fatty acids (gm) were estimated from the first day of foods, beverages, and water (tap and bottled water) consumed from midnight to midnight preceding the medical examination center interview. Information regarding dietary supplements, antacids, and medications are not included in the total dietary nutrient intake variables. Participants with missing data for nutrient intake described were dropped from this study. 

### 2.5. Food Security 

NHANES 2007–2018 provides an adult food security question that places adults within the household into four categories: full; marginal; low; and very low food security. These values were formulated by NHANES from the evaluation of ten questions in the food security questionnaire. The research team dichotomized the values to either secure (full food security) or insecure (marginal, low, and very low food security) to avoid an extreme imbalance in sample sizes between categories. 

### 2.6. Demographics

Additional demographic factors of interest include age, the 6-month time period when the examination was performed, race, marital status, and education. The ‘6-month time period when the examination was performed’ divided the year into 1 November through 30 April (which we labeled winter) and 1 May through 31 October (which we labeled summer). The NHANES race variable divided participants into 5 categories: Mexican American, other Hispanic, Non-Hispanic White, Non-Hispanic Black, and Other Races Including Multiracial. The research team re-coded the variables to combine Mexican Americans and other Hispanic in order to simplify the categorical options. The inclusion of a non-Hispanic Asian category was not a part of the race variable available spanning the 12 years of collected data. The NHANES marital status included 6 categories: married, widowed, divorced, separated, never married, and living with a partner. The research team re-coded the values to combine married participants and those living with a partner. Widowed, divorced, and separated participants were also combined to simplify the categories and produce a more even distribution of sample sizes within each category. Education level in adults 20 years and older evaluated the highest grade level of school completed or the highest degree received. This variable was split into five categories within the NHANES dataset: Less Than 9th Grade, 9–11th Grade (includes 12th grade with no diploma), High School Grad/GED or Equivalent, Some College or AA Degree, and College Graduate or Above. The research team re-coded this variable to combine participants with less than 9th-grade education and those with 9–11th grade education for simplification of the data and a more even distribution of the sample sizes across the categories. Trained health technicians along with a recorder gathered body measurement data which are presented in the body measures section. This study used information regarding body mass index (kg/m^2^) across each of the study populations. 

### 2.7. Statistical Analyses 

All survey observations were weighted to account for population strata and minimize bias. Following NHANES guidelines, the dietary intake survey weights were used for analysis. Since this study used 6 NHANES survey cycles, the NHANES weights were divided by 6, following the recommendations of the NHANES survey instructions. Participants with missing data across the PHQ-9, serum vitamin D concentration, and diet variables of caloric intake, total fats, total saturated fatty acids, total monounsaturated fatty acids, total sugars, total protein, and total carbohydrates were dropped from the analysis. Additionally, survey respondents outside the target age range of 20–44 years old were removed from the dataset prior to analyses. 

The ‘dagitty’ [48] package was used to construct a directed acyclic graph (Figure 1) and identity adjustment sets for estimating the impact of vitamin D on depression severity. Diet was identified as a confounder affecting both depression and vitamin D impact; it was further identified as part of a ‘back door’ path linking depression and vitamin D. Controlling for diet closed that back door path and directly controlled for this confounder. As ‘diet’ is a broad concept representing numerous factors, latent variables for diet were constructed to represent this concept using dietary intake data. Latent variables for diet, “Diet pattern 1” and “Diet pattern 2”, were created by conducting a principal component analysis using total caloric intake, total fats, total saturated fatty acids, total monounsaturated fatty acids, total sugars, total protein, and total carbohydrates. These variables were highly correlated with one another, with the majority of pairwise correlation coefficients being above 0.5, except for correlations between sugars with fat and protein variables. The variables were scaled and standardized to have a mean of zero and a standard deviation of one for principal component analysis. The first two components were extracted and used as control variables representing diet. The R package ‘survey’ [49,50] was used for estimating population weighted-means cross-tabulations and conducting chi-square contingency tests. 

A Bayesian multinomial model was fit to predict depression outcomes for each cohort separately. These outcomes were estimated using cumulative probit and logit models as a function of the natural log of vitamin D and the two latent variables for diet, Diet pattern 1 and Diet pattern 2. The observations were weighted by their NHANES weights, and the model was fit using the R package ‘brms’ [51,52,53] for 4 chains of 2000 iterations each, with 1000 iterations reserved for warmup. Vitamin D was transformed with the natural log to introduce more symmetry into that variable and reduce the influence of extremely large values. The postpartum subpopulation analysis followed a similar form with the following exceptions: (1) separate slopes were estimated for the breastfeeding and non-breastfeeding groups; and (2) unequal variance of the breastfeeding groups was assumed and incorporated into the model. 

Model fit for all populations and subpopulations analyses were evaluated by comparing the WAIC and leave-one-out statistics for the logit and probit models. The convergence and model quality was evaluated for each model by inspecting the trace plots of the chains and the values for R-hat and the effective sample size for each parameter. Post hoc hypothesis tests were conducted to evaluate if the vitamin D slope parameters were less than zero, indicating that as vitamin D concentration increased, the probability of depression decreased. The statistical software R v4.2 or higher was used for all analyses [54].

## 3. Results

### 3.1. Population Characteristics 

**Figure 2:**
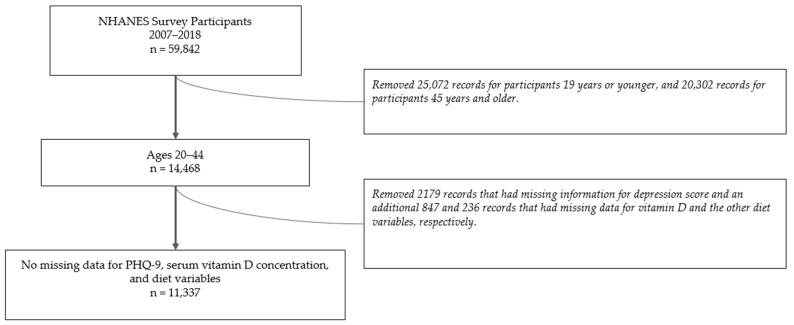
Flow diagram illustrating how we arrived at our final study population after filtering for the desired age range and removing participants with missing data for the PHQ-9, serum vitamin D concentration, and diet variables of caloric intake, total fats, total saturated fatty acids, total monounsaturated fatty acids, total sugars, total protein, and total carbohydrates.

**Figure 3:**
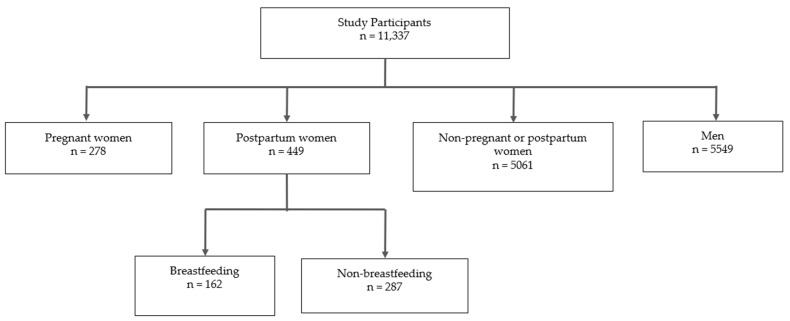
Flow diagram visualizing the count breakdown of participants into subpopulations: pregnant women, postpartum women, non-pp women (non-pregnant/postpartum women), and men and the postpartum breastfeeding and non-breastfeeding subgroups.

**Figure 4:**
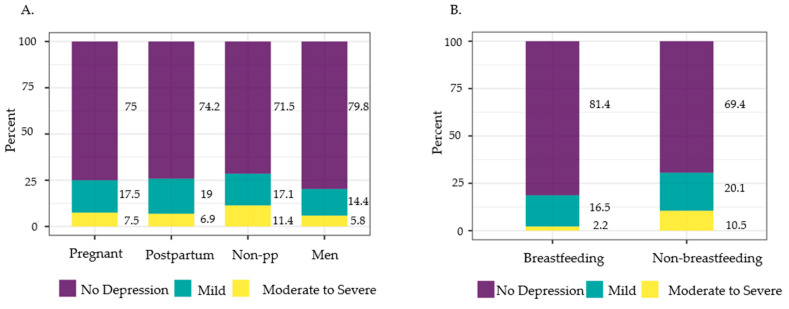
Weighted percentage of participants with no depression, mild depression, and moderate to severe depression in (**A**) pregnant women, postpartum women, non-pp women (non-pregnant/postpartum women), and men; and (**B**) breastfeeding and non-breastfeeding postpartum women aged 20–44 years old, NHANES 2007–2018.

There were 59,842 study participants in NHANES 2007–2018. After filtering for the desired 20–44-year-old age range and dropping participants with missing data in the PHQ-9, serum vitamin D concentration, and diet variables, 11,337 participants remained in this study (Figure 2). A flow diagram (Figure 3) was created to visualize the breakdown of participants into each subpopulation, and the study population characteristics are shown in Table 1. The counts and weighted percentages of depressive symptoms severity for each subpopulation were gathered (Table 2; Figure 4A). Among the pregnant women, postpartum women, non-pp women, and men groups, men exhibited the least weighted percentage (5.8%) in the moderate to severe depression category, while non-pp women had the highest weighted percentage (11.4%). The counts and weighted percentages of depressive symptoms severity for the postpartum breastfeeding and non-breastfeeding women were also noted (Table 3; Figure 4B). Breastfeeding women exhibited the lowest weighted percentage (2.2%) in the moderate to severe depression category of all study groups. 

### 3.2. Latent Variable Analysis

**Figure 5:**
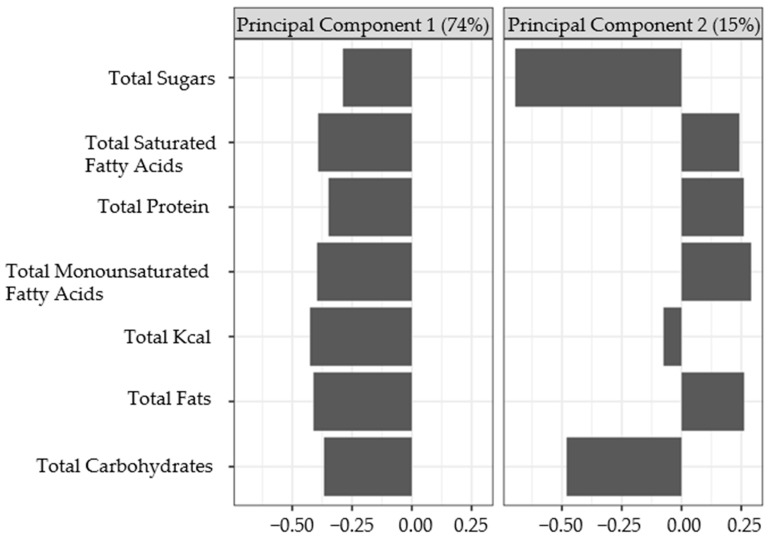
Variable loadings for the diet-derived latent variables from principal component analysis. The percentages in parentheses are the percent variation summarized by each latent variable. The loadings from the first component, a latent variable labeled “Diet pattern 1”, negatively weighted all of the (standardized) Diet pattern 1 variables, where lower values for this latent variable indicated higher overall calorie consumption and elevated consumption of protein, fats, carbohydrates, and sugars—similar to that of a typical Western diet. The second principal component loadings (latent variable “Diet pattern 2”) had negative values for sugar and carbohydrates and positive values for all fats variables and protein, while the loading for total calories hovered close to zero. The higher values for Diet pattern 2 among the respondents for this latent variable indicate more fat and protein consumption and lower carbohydrate and sugar consumption.

The first and second principal components summarized 74% and 15% of the total variation in this population, respectively, for a combined 89% (Figure 5). The loadings from the first component, a latent variable labeled “Diet pattern 1”, negatively weighted all of the (standardized) Diet pattern 1 variables, where lower values for this latent variable indicated higher overall calorie consumption and elevated consumption of protein, fats, carbohydrates, and sugars—similar to that of a typical Western diet. The second principal component loadings (latent variable “Diet pattern 2”) had negative values for sugar and carbohydrates and positive values for all fat variables and protein, while the loading for total calories hovered close to zero. The higher values for Diet pattern 2 among the respondents for this latent variable indicate more fat and protein consumption and lower carbohydrate and sugar consumption. 

### 3.3. Bayesian Cumulative Ordinal Model Estimation

The probit model fit better for all cohorts and the postpartum subgroup analysis according to WAIC and leave-one-out criteria. The R-hat values for all parameters across all models were close to 1; all chains converged, and the effective sample size ratios were within acceptable limits of 0.1 to 1 (most were between 0.35 and 0.75). 

**Figure 6:**
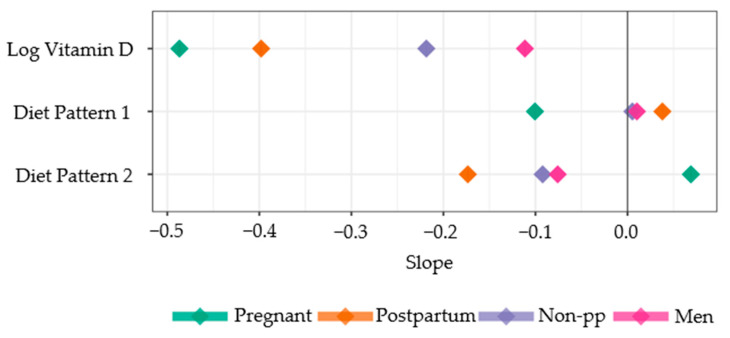
Slope parameter estimates from cumulative categorical model for pregnant women, postpartum women, non-pp women (non-pregnant/postpartum women), and men, using the model threshold and observed data for Diet patterns 1 and 2. The larger negative values for a group correspond to a decreased depression risk. The 68% and 95% credible intervals are plotted but cannot be observed because they are less wide than the plotting symbol for each point.

The latent variable “Diet pattern 1” had a small positive effect (slope) on depression outcomes, corresponding with an increased risk of depression risk for postpartum women (Figure 6). Diet pattern 1 had a larger negative slope corresponding with a decreased depression risk for pregnant women. For postpartum women, the more overall calories and food they consumed, the lower the Diet pattern 1 variable was, resulting in a higher probability of depression. For pregnant women, the opposite was observed. For non-pp women and men, the effect of Diet pattern 1 on depression was near zero. The 99.9% posterior probability intervals for Diet pattern 1 was outside of zero for pregnant and postpartum women only.

The parameter estimates for the effect of Diet pattern 2, indicating higher protein/fat and lower sugar carbohydrate consumption, on depression were negative for postpartum women, non-pp women, and men, and they were positive for pregnant women (Figure 6); the fewer calories, sugar, and carbohydrates consumed and/or greater the protein and fats consumed, the lower the probabilities of depression were in postpartum women, non-pp women, and men. In contrast, pregnant women had increased risk of depression with decreased calories, sugar, and carbohydrate consumption. All 99.9% posterior probability intervals were outside of zero for all cohort estimates. The effects of Diet pattern 2 had absolute values greater than the slopes of Diet pattern 1, indicating that Diet pattern 2 had larger effects on depression than Diet pattern 1 (Figure 6).

**Figure 7:**
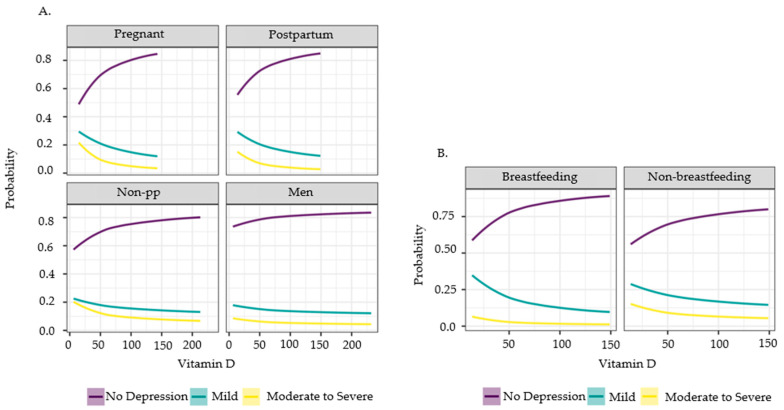
Prediction curve along vitamin D from item response model in (**A**) pregnant women, postpartum women, non-pp women (non-pregnant/postpartum women), and men; and (**B**) breastfeeding and non-breastfeeding postpartum women aged 20–44 years old, NHANES 2007–2018. Predictions are from the observed data for each cohort, taking into account the diet latent variables and population threshold for the depression categories. The 0.999 prediction interval is plotted, but it is narrower than the plotted line and hence not visible.

The estimates for the log vitamin D slope parameter were negative for all cohorts, and zero is outside the 99.9% posterior probability interval for all vitamin D estimates (Figure 6). As vitamin D increased, the probability of “no depression” increased, while the probability of mild or moderate to severe depression decreased. The pregnant cohort had the largest absolute value for vitamin D slope (indicating a steeper change than smaller values for that parameter), followed by postpartum women, then non-pp women and men (Figure 7A). The effects due to vitamin D are greater than those of either diet pattern for every cohort (Figure 6).

For the postpartum subgroup analysis, the effects of the diet latent variables 1 and 2 were 0.00 and −0.11, respectively, which was similar to their results from the “postpartum” cohort analysis. The slope parameters for vitamin D were −0.31 and −0.32 for non-breastfeeding and breastfeeding, respectively (Figure 7B). Their estimated difference, 0.01, was within the 95% credible interval for the hypothesis test as it is different from zero, confirming that these effects are slightly different.

## 4. Discussion

From the 12 years of cross-sectional data collected by the NHANES, we confirmed similar findings from previous publications as well as gathered new information to advance maternal and fetal health. Consistent with previous studies [9,10,11,12,15], vitamin D concentration had a significant negative correlation with depressive symptoms in the pregnant women, postpartum women, non-pp women, and men populations; a higher vitamin D concentration indicates an increased probability of no depression, while a lower vitamin D concentration predicts an increase in the probability of depression. However, the influence of vitamin D concentration on depression differed across the subpopulations. Specifically, this study indicated that vitamin D concentration has a greater impact on pregnant women than the other subpopulations. After pregnant women, postpartum women had the next greatest impact from vitamin D concentration, then non-pp women, and finally men. Although vitamin D concentration impacts men’s depressive symptoms, its effects are lower compared to any of the women subpopulations.

Perinatal depression alters health outcome risks in infants [3,4,5,6,7], and vitamin D concentration was shown to be an influential factor on depression in pregnant and postpartum women. This suggests that adequate vitamin D concentration throughout pregnancy and after delivery may be particularly crucial to the health of the mothers and infants. The literature describes how vitamin D receptors in the central nervous system impact the growth and development of neuronal cells [55,56,57,58]. For example, a mix of human trials and animal models reveal vitamin D influencing orderly brain development [55], neural differentiation [56,57], maturation [57], brain structure and function, and axonal connectivity [56]. Associations have also been noted between vitamin D deficiency in the perinatal period with increased risk of neurodevelopmental disorders, including autism and schizophrenia [56,58]. Furthermore, there is evidence that vitamin D directly impacts serotonin synthesis and indirectly acts as a serotonin reuptake inhibition and monoamine oxidase inhibitor, thus influencing behavioral pathophysiology [59]. Leading into implications of increased serotonin levels, postpartum breastfeeding women display a decreased depression risk with higher serotonin levels [60]. Unfortunately, serotonin levels are not assessed in the continuous NHANES study, and future studies should investigate the relationship between vitamin D, serotonin, and effects on mood.

Our study also analyzed how depression risk among breastfeeding and non-breastfeeding postpartum women are impacted by vitamin D concentration. A novelty found in this study was that the beneficial effects of vitamin D on depression are more enhanced in breastfeeding women than non-breastfeeding women. Specifically, breastfeeding women showed the lowest percentages of moderate to severe depression and highest percentage of participants with no depression compared to all other groups, suggesting that postpartum breastfeeding women are more impacted by the benefits of vitamin D on depressive symptoms compared to non-breastfeeding women. The NHANES reproductive health questionnaire does not specify whether the breastfeeding women are exclusively breastfeeding or mix feeding with formula, but since this group shows the lowest weighted percentage of no depression, at least some breastfeeding activity appears to enhance the benefits of higher vitamin D concentration. This, again, points to the importance of maintaining adequate vitamin D concentration while the women’s bodies are an active source of nutrition for infants, during pregnancy and while breastfeeding.

An important novelty of this work is how it established a link between vitamin D and depression in the cohorts while controlling for important intervening factors. The research team attempted to control for all confounding variables that our literature indicated are germane to understanding vitamin D concentration and depression risk. Diet emerged as a confounding variable and part of a back door confounding path affecting both vitamin D concentration and depression [14,23,24,25]. Diet pattern 1, having higher summative calories and intake of all macronutrients, indicated a positive relationship with depression in postpartum women. Interestingly, Diet pattern 1 closely simulates the typical Western diet, and pregnant women were the only group that exhibited decreased depressive symptoms risk with this diet pattern. Diet pattern 2, which encompassed a lower caloric, sugar, and carbohydrate intake and/or increased protein and fat intake, showed a decreased risk of depression in postpartum women, non-pp women, and men. Pregnant women again echoed another divergence, where following Diet pattern 2 predicted increased risk of depression. Diet pattern 2 has similarities to the paleo diet, and our results show decreased depression risk with this diet for all the study populations, except pregnant women. 

Our study demonstrates how pregnant women need different diet plans than postpartum women, non-pp women, and men to better support their mental health and potential impacts on fetal health. During pregnancy, based on the increased overall caloric and macronutrient needs to support tissue deposition and optimize placental–fetal nutrient transfer, Diet pattern 1 ensures adequate nutritional provision. Indeed, pregnancy induces insulin resistance to maximize glucose transport to the fetus; thus, a diet with higher total calories and overall macronutrients would be conducive to supporting optimal placental–fetal glucose transfer. However, the threshold between the two diet patterns, to avoid an over-abundance of intake inducing obesity risk, and the risk for depression in pregnant women still needs to be investigated. For example, Parrettini et al. reviewed several studies and illustrated how obesity and excessive weight gain presents a higher risk of maternal/fetal health issues in the short and long term [61]. Finding an appropriate nutritional balance for expecting mothers will require a multifactorial approach, assessing body composition, morbidities, and socioeconomic barriers to a healthy diet. It is interesting that postpartum women (including breastfeeding women) did not share the same relationship patterns with pregnant women in relation to diet patterns even though breastfeeding women also require additional caloric intake. However, our subpopulation of postpartum women included both breastfeeding and non-breastfeeding women, and the actual amount of breastfeeding was not clearly quantified in the NHANES data to better assess the impact of these diet patterns on breastfeeding women.

Although our study includes an extensive length of NHANES data, the cross-sectional design does not allow for repeated measures of vitamin D concentrations nor depression scores in individual participants over time. We simply have a snapshot of how vitamin D concentrations are associated with depression risk in pregnant women, but we do not know how this relationship may change over the pregnancy course. Another survey limitation involves the breastfeeding status reported in the reproductive health questionnaire. The question for the NHANES participants only inquires if the woman is currently breastfeeding, not specifying exclusive breastfeeding or actual amount of breastfeeding. As we know, exclusive breastfeeding during the first 6 months of life is recommended for all infants [62], and future studies following mothers could benefit from quantifying exclusive breastfeeding and amount of breastfeeding to better understand human milk’s protective effects. Additionally, our sample sizes for participants with moderate to severe depression were limited, and our pregnant/postpartum groups were much smaller than our non-pp women and men groups. A larger pool of pregnant and postpartum participants and participants experiencing depression would strengthen the results of subsequent studies.

Future research analyzing how human milk composition changes with varying vitamin D concentrations in women may provide important health knowledge for breastfed infants. Future studies should also investigate whether vitamin D concentrations are significantly different geographically throughout the United States; vitamin D concentration is affected by endogenous synthesis through sun exposure, and populations across the US range in the amount of exposure throughout the year. Building on our newfound knowledge from this study that pregnant and postpartum women display stronger influences from vitamin D concentration, this may inform interventional studies throughout the perinatal period in relation to maternal mental health.

## 5. Conclusions

This 2007–2018 NHANES cross-sectional study illuminates to readers that higher vitamin D concentration decreases depression risk more significantly in pregnant and postpartum women compared to non-pp women and men. Among postpartum women, vitamin D concentration had a greater influence on decreasing breastfeeding women’s depression risk than non-breastfeeding women. Through the diet pattern analysis, postpartum women revealed a positive relationship with a typical Western diet and depression, while non-pp women and men showed a negligible association. Postpartum women, non-pp women, and men all showed decreased depression risk with a diet similar to the paleo diet. Pregnant women were shown to deviate from postpartum women, non-pp women, and men by having decreased depression risk with a typical Western diet and increased depression risk in a diet similar to the paleo diet.

## Figures and Tables

**Table 1:** Weighted percentages and distributions (mean ± standard deviation) of characteristics among all participants, pregnant women, postpartum women, non-pp women (non-pregnant/postpartum women) and men aged 20–44 years old, NHANES 2007–2018.

Variable Description	All Participants	Pregnant	Postpartum	Non-pp ^1^	Men
Race					
Non-Hispanic White	58.7	51.2	55.1	59.3	58.8
Hispanic	19.5	21.2	23.9	18.3	20.1
Non-Hispanic Black	12.6	15.2	13.6	13.5	11.7
Other Race—Including Multiracial	9.2	12.3	7.4	9	9.4
Marital Status					
Married/Living with Partner	56.7	78.1	75.2	56.2	54.7
Widowed, Divorced, or Separated	8.5	5	6.5	11.3	6.4
Never Married	34.8	16.9	18.3	32.5	38.9
Education					
No High School Diploma	14.1	17.4	17.1	12.7	15
High School Grad/GED or Equivalent	22.3	18.9	25.3	18.8	25.2
Some College or AA Degree	34.4	32.7	31.9	36.9	32.6
College Graduate or Above	29.1	31	25.6	31.5	27.3
Adult Food Security Category					
Secure	68.7	67.7	62.2	67.5	70.3
Insecure (Marginal to Very Low)	31.3	32.3	37.8	32.5	29.7
Data Collection Season					
Summer	55.8	59.8	52.9	56.3	55.5
Winter	44.2	40.2	47.1	43.7	44.5
Age	31.7 ± 7.2	29.1 ± 5.9	28.4 ± 5.8	32.2 ± 7.4	31.5 ± 7.2
BMI	28.7 ± 7.5	29.9 ± 7.1	29.7 ± 7.5	28.9 ± 8.3	28.5 ± 6.6
Income: Poverty Ratio	2.7 ± 1.6	2.8 ± 1.6	2.1 ± 1.4	2.7 ± 1.6	2.8 ± 1.6

^1^ Non-pp, non-pregnant/postpartum women.

**Table 2:** Counts of depression level in pregnant women, postpartum women, non-pp women (non-pregnant/postpartum women), and men aged 20–44 years old, NHANES 2007–2018.

Depression Level	Pregnant	Postpartum	Non-pp ^1^	Men	Total
None	194	336	3570	4409	8509
Mild	60	83	897	790	1830
Moderate to severe	24	30	594	350	998
Total	278	449	5061	5549	11,337

^1^ Non-pp, non-pregnant/postpartum women.

**Table 3:** Counts of depression level in breastfeeding and non-breastfeeding women aged 20–44 years old, NHANES 2007–2018.

Depression Level	Breastfeeding	Non-Breastfeeding	Total
None	127	209	336
Mild	29	54	83
Moderate to severe	6	24	30
Total	162	287	449

## Data Availability

The raw data supporting the conclusions of this article will be made available by the authors on request.
